# Roughness-dependent retention of a reduced rabies-virus model in a platinum nanochannel: a molecular dynamics study

**DOI:** 10.1016/j.virusres.2026.199816

**Published:** 2026-10-08

**Authors:** Narinderjit Singh Sawaran Singh, Murtaja Satea, Ali Jawad Kadhum, Ibrahm Mahariq, Hemn A.H. Barzani, Baydaa Abed Hussein, Mina D. Jumaah, Mahmut Taner, Soheil Salahshour, A. Rahimi

**Affiliations:** a Faculty of Data Science and Information Technology, INTI International University, Persiaran Perdana BBN, Putra Nilai, Nilai, 71800, Malaysia; b College of Medicine, University of Warith Al-Anbiyaa, Karbala, 56001, Iraq; c Department of Biology, College of Science, University of Al Maarif, Al Anbar, 31001, Iraq; d University College, Korea University, Seoul, 02481, South Korea; e Department of Medical Research, China Medical University Hospital, China Medical University, Taichung, Taiwan; f Department of Medical Laboratory Science, College of Health Science, Lebanese French University, Erbil, Iraq; g Department of pathological analyzes, University of Manara, Maysan, Iraq; h College of Medicine, Misan University, Maysan, Iraq; i Faculty of Engineering and Architecture, Istanbul Gelisim University, Istanbul, Türkiye; j Faculty of Engineering and Natural Sciences, Istanbul Okan University, Istanbul, Türkiye; k Faculty of Engineering and Natural Sciences, Bahcesehir University, Istanbul, Türkiye; l Research Center of Applied Mathematics, Khazar University, Baku, Azerbaijan; m Faculty of Science and Letters, Piri Reis University, Tuzla, Istanbul, Türkiye; n Fast Computing Center, Shabihsazan Ati Pars, Tehran, Iran

**Keywords:** Reduced rabies-virus model, Molecular dynamics, Nanochannel, Surface roughness, Nanoscale confinement, Infection disease

## Abstract

Capturing nanoscale biological objects in nanochannels depends strongly on surface geometry and interfacial interactions. However, the spatial and temporal limitations of atomistic simulations prevent the direct representation of a complete rabies virion. In this study, we used molecular dynamics simulations to examine the force-driven retention of a reduced rabies-virus model with a characteristic size of approximately 4 nm in a water-filled platinum nanochannel with non-ideal surface roughness. The simulated construct was developed from reported rabies-virus structural information but did not reproduce the complete morphology, envelope, genomic content, or biological functionality of an intact virion. The atomic interactions were described using DREIDING and Universal Force Field parameters, the TIP3P water model, the embedded atom method for Pt–Pt interactions, and Lennard-Jones and Coulombic terms for interfacial interactions. After 10 ns of canonical equilibration at 300 K, the system underwent a 20 ns force-driven production stage. Roughness sizes of 35, 37, 40, and 43 Å were investigated. Among the four simulated configurations, the 40 Å roughness case produced the largest maximum density (0.187 atom/Å³), the lowest maximum velocity (0.006 Å/ps), the largest retained surface-mesh volume (31,118 Å³), and the most negative reduced-model–Pt interaction energy (−469.91 kcal/mol). The simulation-defined retained fraction for the reduced construct reached 95% at 20 ns. The weaker responses at 43 Å indicated a non-monotonic dependence on roughness size within this model and the investigated geometries. These results characterized the force-driven behavior of a specific 4-nm construct under the stated simulation conditions; they did not establish roughness-dependent capture behavior for intact rabies virions or predict experimental device performance.

## Highlights

• For various medical purposes, virus trapping in biosystems is essential.
• Nanochannels can be implemented for this purpose appropriately.
• Simple virus (Rabies) in an aqueous environment is trapped by a non-ideal nanochannel.
• MD outputs indicated atomic stability of defined systems in the presence of water molecules.
• Virus trapping efficiency converged to 95% by atomic roughness size optimizing after t=20 ns.

## 1 Introduction

Viral diseases represent and will remain a significant issue for global public health with a range of transmission routes, ongoing evolution, and the potential to cause significant morbidity and mortality (Saldaña et al., 2024; Hashim et al., 2025). Effective management of viral infections depends not only on therapeutic and vaccination strategies, but also on sensitive detection and, for many analytical platforms, the ability to capture, enrich, isolate, and characterize viral particles, particularly when their abundance in the analyzed sample is low (Guo et al., 2024; Lopez et al., 2024). Rabies is a special case in that it is inevitably fatal after the onset of clinical signs, and there is degeneration of the nervous system which spreads relentlessly (Bano et al., 2017). Consequently, rapid identification of viral particles and development of efficient approaches for their physical separation and capture remain important components of diagnostic and biosensing research (Shiohara et al., 2023). Given the specific challenges at the nanoscale, interest has grown in nanostructured devices that can manipulate biomolecules through confinement, interfacial interaction, electrostatic interaction, and size-filtering transport (Hu et al., 2025).

Recent developments in viral diagnostics demonstrated that nanoscale materials can considerably improve the sensitivity and selectivity of conventional detection platforms (Gataa et al., 2025). For instance, Zeng et al. (Zeng et al., 2025) also constructed a signal-enhanced lateral-flow immunoassay using core-shell quantum dots for highly sensitive protein detection, showing the efficacy of nanostructured labels in improving sensitivity for bioanalyses. Beyond conventional biochemical analyses, advances in medical Internet-of-Things technologies opened new ways to acquire, transmit, and process biomedical information safely. For example, Jin et al. (Jin et al., 2025) proposed a dynamic encryption scheme for the medical Internet of Things, highlighting the combined trend of sensing, computation, and secure medical data storage. It showed that future diagnostic systems combined nanoscopic capture/recognition principles with intelligent downstream data-processing technologies.

The biological relevance of virus-related technologies extended beyond direct detection because infection, vaccination, and host immune responses were strongly interconnected (Alkhafaji et al., 2026). Recent studies demonstrated that vaccine-induced immune activation can occasionally produce complex systemic responses. Jia et al. (Jia et al., 2023) systematically evaluated reported cases of Sweet syndrome following severe acute respiratory syndrome coronavirus 2 vaccination and discussed possible links with innate immune activation. Xu et al. (Xu et al., 2023) similarly investigated the clinical characteristics, diagnosis, and management of vaccine-associated Vogt-Koyanagi-Harada disease. Zhang et al. (Zhang et al., 2023) examined reported cases of coronavirus disease vaccine-induced hemophagocytic lymphohistiocytosis, further illustrating the multifaceted nature of immune responses to viral antigens and vaccination. While these works focused on clinical immunology instead of virus confinement, they illustrated the general biomedical imperative of precise viral detection and the interaction between biological systems and engineered diagnostic and therapeutic platforms.

Nanotechnology provided an important bridge between such biological requirements and engineering-based virus management. The sizes of nanoparticles, nanostructured interfaces, nanopores, and nanochannels were comparable to those of many biological macromolecules and can therefore lead to significant confinement and surface-interaction effects. Wang et al. (Wang et al., 2024) recently developed macrophage-derived biomimetic nanoparticles for light-driven theranostics against Mpox. Their findings suggest that nanoscale materials can be designed as functional interfaces that not only passively host viruses but also interact specifically with the biological context surrounding the virus. Related studies on naturally derived compounds and molecular bioactivity illustrated the continuing search for molecular structures with biologically relevant interactions (Chen et al., 2019). Collectively, these investigations demonstrated that controlling interactions at biological interfaces was increasingly important for virus detection, treatment, and nanomedicine.

Nanostructured platforms, particularly nanochannels, were of particular interest because their confined dimensions enable control of particle transport through steric confinement, surface forces, local hydrodynamics, and physicochemical interactions (Hu et al., 2025). Indeed, recent advances in nanochannel electrochemical sensing suggest that nanochannels can enhance selective transport and surface interfacial recognition events, which are key elements of highly sensitive sensing systems. The interplay among channel geometry, surface topography, and transported species was particularly interesting because small nanoscale departures from an ideal smooth surface can significantly alter local fluid flow paths and particle-surface interactions. Surface roughness may create a more favorable adsorption area, increase contact likelihood, modify local residence times, and therefore alter the likelihood that a carried biological species stays in the nanochannel. As a functional parameter, surface roughness should be included in a device design.

Experimental characterization of these mechanisms was challenging because many events responsible for nanoscale trapping occur over molecular dimensions and very short time scales. Molecular dynamics (MD) simulation provided a useful complementary approach because it can resolve atomic trajectories directly, and quantities such as interaction energy, density distribution, molecular velocity, temperature (T), radial distribution functions (RDF), and particle displacement can be determined from simulated trajectories (Xiao et al., 2024; Alkhafaji et al., 2026). Previous MD investigations demonstrated the sensitivity of confined systems to channel geometry and interfacial structure. Zhang et al. (Zhang et al., 2023) investigated how nanochannel size and structural characteristics affected confined materials and showed that modifying nanoscale channel geometry can substantially change atomic organization and transport behavior. MD studies by Cai et al. (Cai et al., 2022) analyzed molecular behavior around metallic interfaces and showed that solid-fluid interfacial interactions significantly affect localized structural and transport properties. These studies focused on nonbiological systems but laid the foundation for atomic-level studies of how solid-fluid interactions and solid geometry regulated nanoscale transport.

Despite these advances, the microscopic mechanisms governing virus retention inside non-ideal nanochannels remained insufficiently understood. Most virus-detection studies focused on biochemical recognition, optical signals, electrochemical responses, or macroscopic capture efficiency, whereas the atomic-scale evolution of a virus-like biological structure during confinement received considerably less attention. Relatively little information exists on how atomistic-scale surface roughness affects virus-surface interaction, molecular arrangement, translation, and residence inside a water-filled nanochannel, or on the optimal roughness, assuming greater surface irregularity leads to higher binding affinity. However, large geometrical irregularities may not be preferable, as they may reduce confined-space volume or disrupt fluid dynamics; they may also create a geometrical barrier to an incoming particle or its transport. A molecular approach was needed to correlate roughness dimensions with confinement behavior.

For these reasons, the present study investigated how rectangular atomistic surface roughness affected the force-driven motion and retention of a reduced rabies-virus model with a characteristic size of approximately 4 nm in a water-filled platinum nanochannel. The molecular construct was developed using reported rabies-virus structural information, but it was not intended to reproduce the dimensions, morphology, envelope, genomic content, or complete biological complexity of an intact rabies virion. Surface roughness was evaluated as an atomistic design parameter by examining changes in spatial distribution, translational mobility, interaction energy, retained volume, and residence within the roughness region. Accordingly, the analysis aimed to identify comparative molecular mechanisms among the simulated geometries rather than predict the quantitative capture efficiency of a full-size rabies virion.

The resulting molecular information provides a mechanistic basis for understanding how surface irregularities can alter the confinement of reduced nanoscale biological models. These comparative trends may support design hypotheses for nanostructured capture, separation, and biosensing platforms. However, the dimensions and biological complexity of an intact rabies virion substantially exceed those of the present 4-nm construct. Therefore, the reported roughness-dependent responses should be treated as atomistic proof-of-concept observations that require multiscale modeling and experimental validation before applying them to full-size viruses or practical nanofluidic devices.

## 2 Computational method

MD simulations were performed using Large-scale Atomic/Molecular Massively Parallel Simulator (LAMMPS, Version 2020) (Plimpton, 1995; Plimpton and Thompson, 2012; Plimpton et al., 1997) to investigate how atomic-scale surface roughness affected the force-driven retention of a reduced rabies-virus model in a water-filled platinum nanochannel. The simulated biological construct had a characteristic size of approximately 4 nm and served as a computational probe of confinement rather than a dimensionally complete representation of a natural rabies virion. Avogadro and Packmol were used to prepare the molecular structure and initial simulation system (Hanwell et al., 2012; Martínez et al., 2009). The computational procedure included system construction, definition of atomic interactions, thermodynamic equilibration, force-driven transport, and trajectory-based characterization of the reduced model.

### 2.1 System construction and simulation model

The simulated system consisted of a reduced rabies-virus model, water molecules, and a non-ideal nanochannel constructed from Pt atoms. The biological component had a characteristic size of approximately 4 nm and was developed using the structural information reported previously (Albertini et al., 2008; Riedel et al., 2019). Because this construct was substantially smaller and structurally simpler than an intact rabies virion, we used it only to examine the molecular effects of hydration, confinement, surface contact, and roughness-dependent retention. It did not reproduce the complete bullet-shaped morphology, envelope, surface-protein distribution, genomic content, or biological functionality of a natural virion. The complete system was placed in a rectangular simulation box (100 × 100 × 200 Å³), and periodic boundary conditions were applied in all three spatial directions, as shown in Fig. 1. The aqueous phase was represented by explicit three-site TIP3P water molecules surrounding the reduced viral model. The Pt nanochannel had dimensions of 100 × 100 × 75 Å³ and contained four Pt atoms per unit cell. Rectangular surface irregularities were introduced into the Pt wall to represent atomic-scale roughness. Roughness sizes of 35, 37, 40, and 43 Å were investigated while the other modeled parameters were held constant. This comparison tests how roughness size affected the motion, surface contact, and retention of a particular 4-nm construct in the simulated channel. Because the construct lacked the complete virion morphology, envelope, glycoprotein distribution, and genomic components, the comparison could not establish how an intact rabies virion would respond to these roughness geometries.

**Fig. 1:**
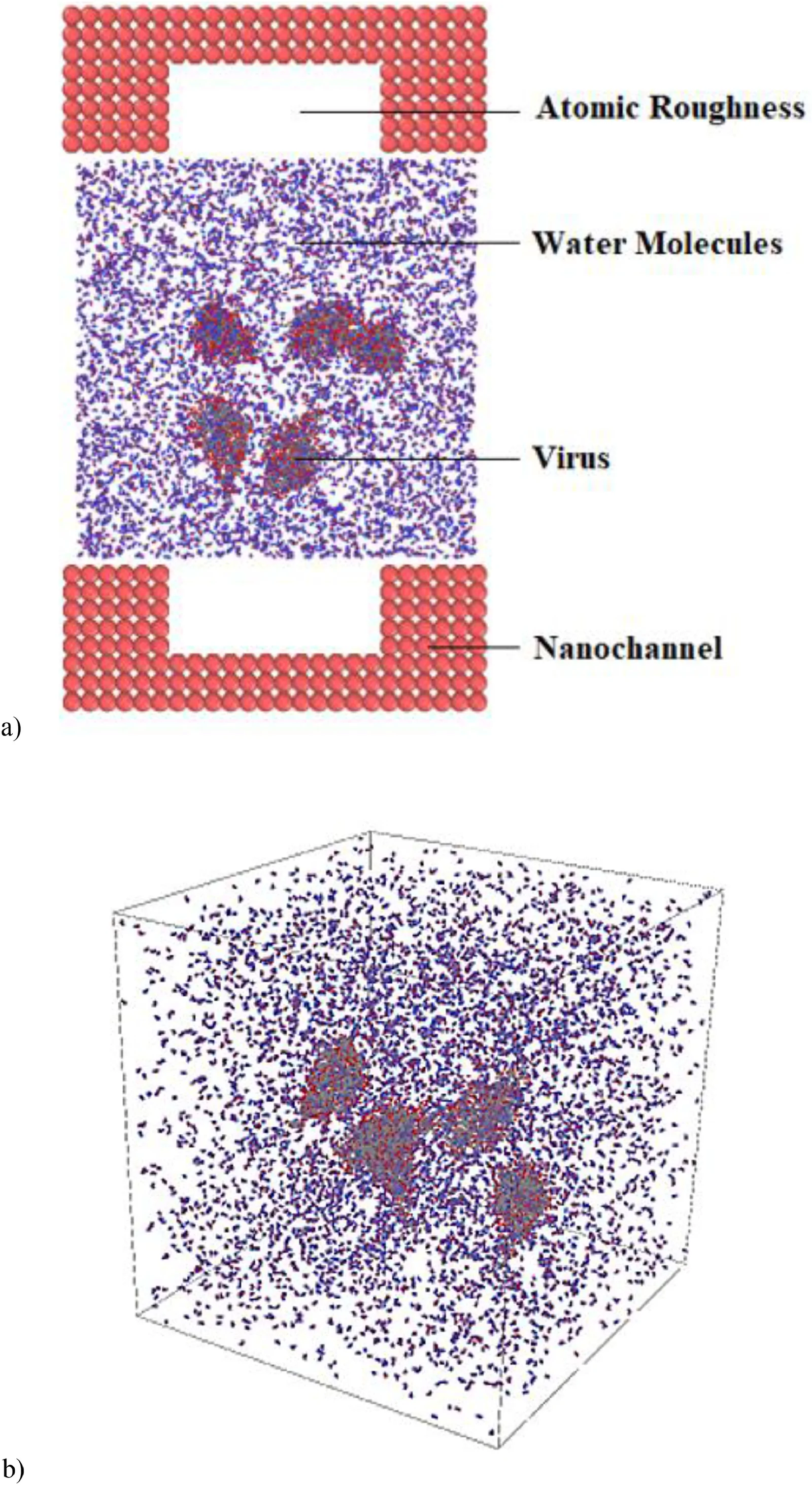
Atomic structures of (a) the reduced 4-nm rabies-virus-model–H₂O–nanochannel system inside the simulation box and (b) the reduced rabies-virus-model–H₂O system.

### 2.2 Force fields and interatomic interactions

The accuracy of an MD simulation depended strongly on potential-energy functions used to represent bonded and non-bonded atomic interactions. In the present system, DREIDING and Universal Force Field descriptions, the TIP3P water model, and the embedded atom method were used for different components of the reduced-model–H₂O–Pt nanochannel system (Rappé et al., 1992; Mayo et al., 1990; Jorgensen et al., 1983; Daw and Baskes, 1984). These classical interaction models were used to compare the confinement behavior of reduced constructs and were not intended to reproduce the full molecular complexity of an intact virion. Non-bonded van der Waals interactions involving the molecular components were described using a 12–6 Lennard-Jones (LJ) potential (Eq. (1)) (Jones, 1924),

(1) $$\phi \left(r_{ij}\right)=4\varepsilon \left[{\left(\frac{\sigma }{r_{ij}}\right)}^{12}-{\left(\frac{\sigma }{r_{ij}}\right)}^{6}\right]\;\;r_{ij}\leq r_{c}$$

where ε represents the depth of the potential-energy well, σ is the characteristic distance at which the LJ potential becomes zero, and *r*ᵢⱼ is the distance between interacting atoms *i* and *j*. Table 1 summarizes elemental LJ parameters used in the simulations. A cutoff radius of 12 Å was applied to LJ interactions. This cutoff was selected because the dispersion contribution decreases rapidly with intermolecular distance according to *r*⁻⁶ term and becomes relatively small at sufficiently large separation (Rappé et al., 1992; Mayo et al., 1990). We retained the same cutoff throughout the simulations to provide a consistent numerical description of relevant short-range non-bonded interactions (Klauda et al., 2007). Bond stretching within the molecular structures was represented using a harmonic potential (Eq.(2)) (Hayek, 2003; Tipler and Mosca, 1999):

(2) $$E_{simple}=1/2k_{r}{\left(r-r_{0}\right)}^{2}$$

where *k*ᵣ is the bond force constant, *r* is the instantaneous bond length, and *r*_₀_ is the corresponding equilibrium bond length. Bond-angle deformation was similarly described by a harmonic angular potential (Eq.(3)):

(3) $$E_{angular}=1/2k_{\theta }{\left(\theta -\theta_{0}\right)}^{2}$$

where *k*_θ_ is the angular force constant, θ represents the instantaneous bond angle, and θ_₀_ is the equilibrium angle. The corresponding bonded parameters were assigned according to the DREIDING force field. Water molecules were represented using the TIP3P model. In this three-site representation, partial atomic charges were assigned to the oxygen and hydrogen sites, with a negative partial charge of 0.834 e assigned to the oxygen atom and a positive partial charge of 0.417 e assigned to each hydrogen atom. TIP3P model was selected because of its computational efficiency, compatibility with conventional all-atom biomolecular MD simulations, and suitability for systems containing large numbers of explicit water molecules.

**Table 1:** LJ parameters employed for the different atomic species in the MD system (Rappé et al., 1992).

Atom type	σ (Å)	ε (kcal/mol)
**N**	3.660	0.069
**C**	3.851	0.105
**H**	2.886	0.044
**O**	3.500	0.060
**Pt**	2.754	0.080

Electrostatic interactions among charged atomic sites were evaluated according to the Coulomb potential (Eq.(4)):

(4) $$U_{C}\left(r_{ij}\right)=\frac{1}{4\pi \varepsilon {}_{0}}\frac{q_{i}q_{j}}{r_{ij}}$$

where qᵢ and qⱼ denote the partial charges assigned to atoms i and j, respectively, ε₀ is the vacuum permittivity, and rᵢⱼ is their separation distance. The partial charges assigned to the atoms of the modeled viral structure were obtained from the ACC online calculator. The electrostatic interactions were treated using Coulomb's relation, with a real-space Coulomb cutoff of 10 Å.

Interactions among Pt atoms constituting the nanochannel were represented using an EAM potential, which accounts for the many-body character of metallic bonding. The total EAM energy can generally be expressed as (Eq. (5)):

(5) $$E=\sum_{i}F_{i}(\rho_{i})+\frac{1}{2}\sum_{i}\sum_{j\neq i}\phi_{ij}\left(r_{ij}\right)$$

where *F*_i_ is the embedding energy of atom *i* as a function of local electron density ρ_i_, and φ_ij_ represents the pair contribution between Pt atoms *i* and *j*. Because the EAM potential described Pt–Pt interactions but did not independently define Pt–water or Pt–virus interactions, the cross interactions between Pt atoms and the molecular species were represented through the LJ description using the Pt parameters reported in Table 1.

The Pt–O, Pt–H, Pt–C, and Pt–N cross-interaction parameters were determined using the default mixing rule in the LAMMPS package used in the simulations. These mixing rules are implemented as follows (Eqs. (6) and (7)),

(6) $$\sigma_{ij}=\sqrt{\sigma_{i}\sigma_{j}}$$

(7) $$\varepsilon_{ij}=\sqrt{\varepsilon_{i}.\varepsilon_{j}}$$

In these relations, i and j represent the types of elements that interacted. Accordingly, the resulting σᵢⱼ and εᵢⱼ values for Pt–O, Pt–H, Pt–C, and Pt–N were calculated by assuming a 2.754 Å and 0.080 kcal/mol ratio for sigma and epsilon parameters for Pt atoms as listed in Table 2. We explicitly defined these cross-interaction parameters through the pair_style and pair_coeff command/settings in LAMMPS and kept them unchanged throughout the simulations (Simulator, Molecular Massively Parallel, 2003).

**Table 2:** LJ parameters employed for the different cross-interaction parameters in the modeled MD system in the LJ formalism (Rappé et al., 1992).

Interaction type	σ_ij_ (Å)	ε_ij_ (kcal/mol)	Atom type	σ_ij_ (Å)	ε_ij_ (kcal/mol)
**N-C**	3.754	0.085	**C-H**	3.334	0.068
**N-H**	3.250	0.055	**C-O**	3.671	0.079
**N-O**	3.579	0.064	**C-Pt**	3.257	0.092
**N-Pt**	3.175	0.074	**H-O**	3.178	0.051
**O-Pt**	3.105	0.069	**H-Pt**	2.819	0.059

### 2.3 Simulation protocol and numerical settings

The reduced-model–H₂O–Pt system was initially equilibrated in the canonical ensemble (NVT) at 300 K for 10 ns. T was controlled using a Nosé–Hoover thermostat with a damping parameter of 10 fs (Tipler and Mosca, 1999; Simulator, Molecular Massively Parallel, 2003; Verlet, 1967). No barostat was used during this stage because pressure-controlled equilibration caused instability in the confined model. Atomic trajectories were integrated using the velocity-Verlet algorithm (Verlet, 1967; Press et al., 2007; Hairer et al., 2003) with a timestep of 0.1 fs. This stage relaxed the initial molecular configuration and established a thermally stable state before the force-driven retention simulation. After 10 ns of canonical equilibration, we removed the thermostat and propagated the system for an additional 20 ns using microcanonical (NVE) integration (Hilbert et al., 2014). During this production stage, we applied a constant external force of 0.01 kcal/(mol·Å) in the X direction to the water molecules and the atoms of the reduced viral model using the LAMMPS addforce command. The force was applied throughout the 20 ns production run to drive the reduced construct through the nanochannel and examine its subsequent confinement. Thus, 10 ns and 20 ns periods represented two physically distinct stages: thermodynamic equilibration and force-driven production, respectively. The resulting trajectories described the nonequilibrium transport of a reduced 4-nm model within the finite nanochannel and did not represent the hydrodynamic transport of a full-size rabies virion. We performed all MD simulations using the described method and repeated each 5 times; we report average values.

### 2.4 Model limitations

The present MD simulations were subject to several limitations that define the scope of reported findings. First, the accessible timescale was restricted to 10 ns of equilibration followed by 20 ns of force-driven production. This duration allowed examination of short-timescale hydration, interfacial contact, molecular transport, and confinement within the simulated system, but it was insufficient to represent macroscopic virus transport, rare retention events, or the long-term performance of an experimental device. Second, the biological object was represented by a reduced rabies-virus model with a characteristic size of approximately 4 nm. This construct was considerably smaller and structurally less complex than an intact natural rabies virion. It did not reproduce the full virion dimensions, bullet-shaped morphology, lipid envelope, complete distribution of surface glycoproteins, genomic content, or biological activity. Consequently, the calculated retained fraction, volume, velocity, density, T, and interaction energies applied only to this reduced model. These quantities must not be interpreted as quantitative estimates of capture efficiency or binding behavior of a full-size rabies virion. Third, the interactions were represented using classical force fields, including DREIDING and Universal Force Field terms for the molecular structure, TIP3P for water, and an embedded atom method potential for Pt–Pt interactions. These descriptions did not explicitly account for electronic polarization, charge transfer, chemical reactions, or bond breaking and formation. The reported interaction energies therefore represented effective classical energetic descriptors of a reduced model, not electronically resolved binding free energies for an intact virus. Finally, the Pt nanochannel wall was maintained using a fixed-geometry approximation. This treatment isolated the effect of surface topography but excluded Pt lattice vibration, surface reconstruction, deformation, and other dynamic interfacial responses. Accordingly, these results establish comparative atomistic trends among the examined roughness geometries. Extending these findings to natural rabies virions or fabricated capture devices requires larger-scale models, longer simulations, deformable surfaces, independent replicas, and experimental validation.

## 3 Results and discussion

### 3.1 Equilibration and structural stability

Before examining the force-driven retention of the reduced model, the thermal and energetic stability of the reduced-model–H₂O–nanochannel system was assessed during a 10 ns canonical (NVT) equilibration. This step ensured the system reached a thermodynamically stable configuration before applying the external driving force. Fig. 2a presents the temporal evolution of T, confirming that the thermostat maintained the system at the prescribed 300 K throughout the equilibration interval. The absence of systematic T drift indicated that the system reached a stable thermal state. Fig. 2b shows the corresponding potential energy, which decreased during the initial relaxation period and plateaued after approximately 3 ns, remaining nearly stationary thereafter. For the 35 Å roughness configuration, the potential energy reached approximately −2219.72 kcal/mol at the end of the 10 ns stage. The initial decrease reflected relaxation of atomic coordinates from the constructed geometry toward lower-energy configurations, driven by optimization of interatomic distances and the formation of favorable hydration contacts. Once the system settled into a locally stable arrangement, the potential energy changed little. The convergence of both T and potential energy therefore confirmed that the reduced computational system reached thermal and energetic equilibrium under the adopted conditions.

**Fig. 2:**
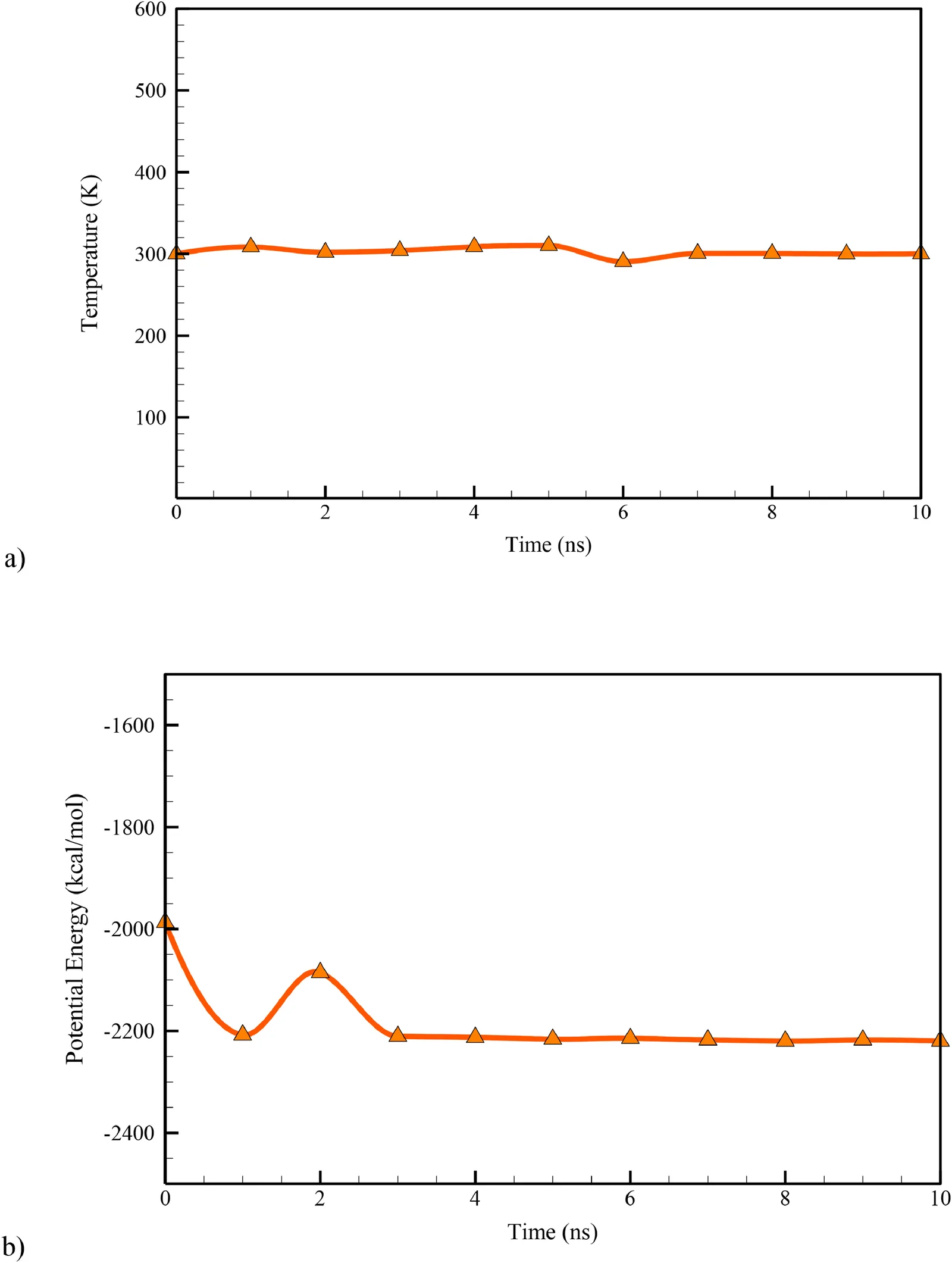
Temporal evolution of (a) T and (b) potential energy of the reduced rabies-virus-model–H₂O–nanochannel system during the 10 ns canonical equilibration stage.

The structural quality of the aqueous phase surrounding the reduced model was further examined using the oxygen–oxygen radial distribution function (O–O RDF), shown in Fig. 3. The RDF characterized the local ordering of water molecules around the reduced construct and served as a diagnostic of the equilibrated liquid state. The pronounced first peak corresponded to the first hydration shell, reflecting short-range O–O correlations at hydrogen-bonding distances. The subsequent minimum separated the first and second coordination regions, while at larger distances the RDF approached unity with progressively weakening oscillations, the signature of a disordered liquid with no long-range positional order. This behavior was consistent with the well-established structural characteristics of liquid water described by the TIP3P model and agreed closely with the reference system (Clark et al., 2010). The RDF profile therefore confirmed that an equilibrated aqueous environment surrounded the reduced model before the force-driven production stage. Together, the potential-energy plateau and the physically expected RDF profile provide two consistent indicators of thermodynamic equilibration.

**Fig. 3:**
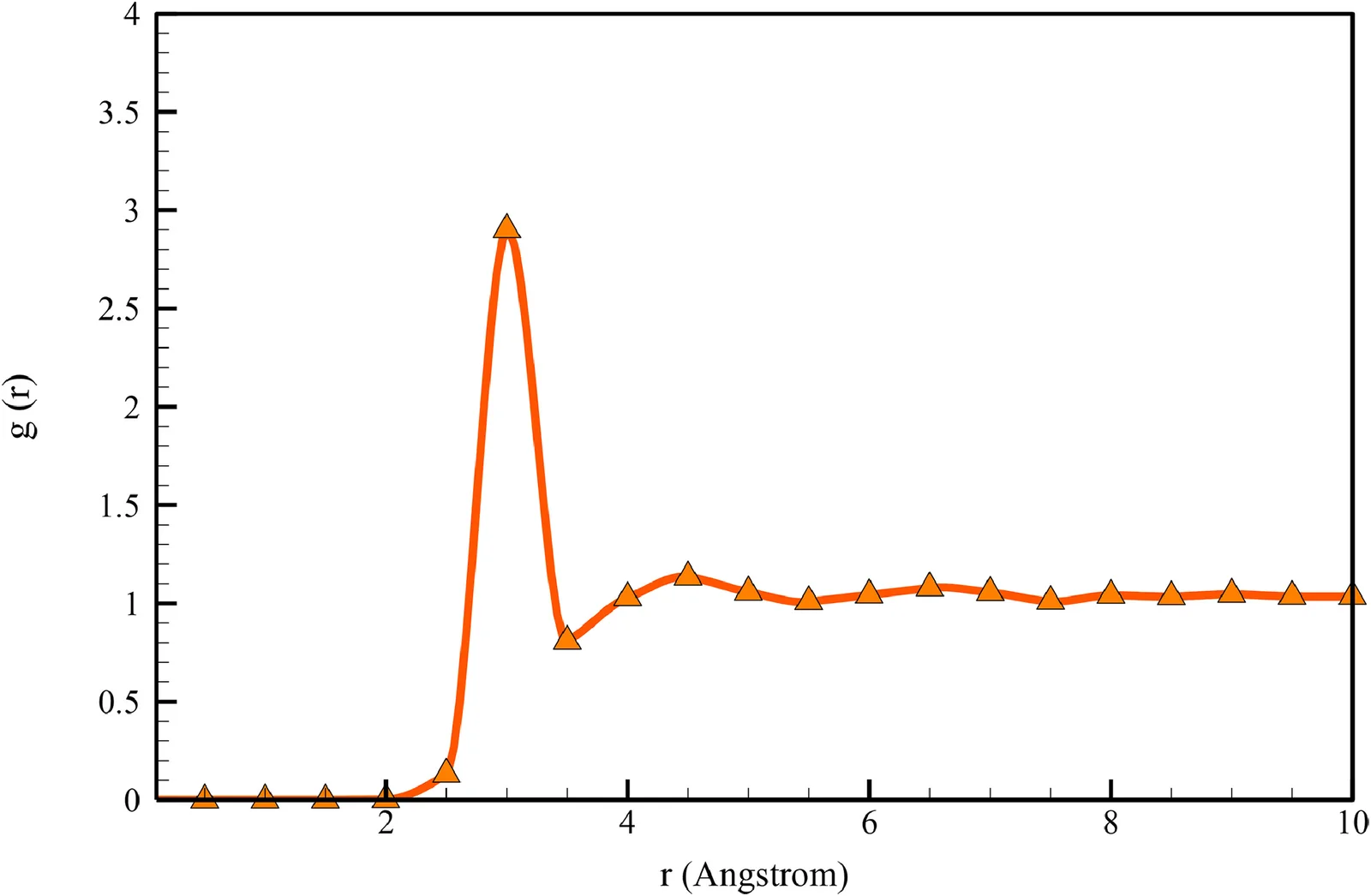
RDF of oxygen atoms in the aqueous phase surrounding the reduced model after NVT equilibration.

### 3.2 Density, velocity, and temperature profiles during retention of the reduced model

Following equilibration, the thermostat was removed, and an external force was applied to the water molecules and the reduced model, initiating a 20 ns force-driven nonequilibrium production stage. The external force drove the reduced construct through the nanochannel toward the rough Pt region, where geometric wall irregularities impede further translational motion. To characterize this process, the simulation box was divided into 60 bins along the Z direction, and local density, velocity, and T profiles were evaluated as complementary descriptors of the reduced construct's spatial localization and kinetic response. Fig.4a represents the schematic representation of the external driving force applied to the H₂O–reduced-model system during the production stage. Fig. 4b presents the density profile obtained after 20 ns of force-driven production for the 35 Å roughness configuration. The maximum local density increased from 0.174 atom/Å³ before retention to 0.181 atom/Å³ after retention. This increase indicated atom accumulation within the confined rough region, where the geometric features of the Pt wall restricted the available volume and promoted spatial localization of the reduced construct. The density enhancement alone provided an initial indication of localization; therefore, it was examined alongside the velocity, structural, volumetric, and energetic descriptors to obtain a comprehensive picture of retention behavior.

**Fig. 4:**
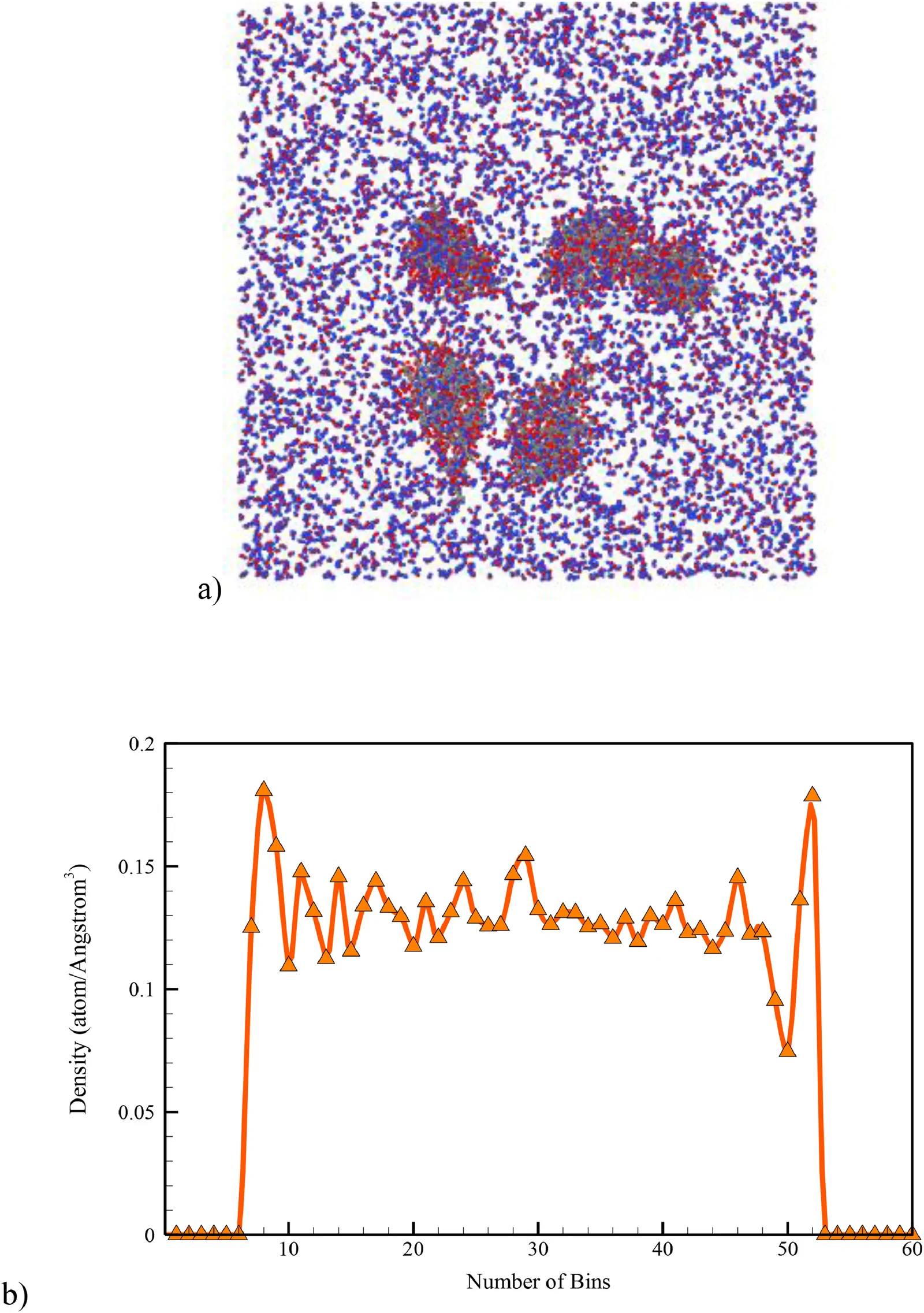
(a) Schematic representation of the external driving force applied to the H₂O–reduced-model system during the production stage and (b) its density profile after 20 ns.

Fig. 5 shows the velocity profile of the H₂O–reduced-model system after 20 ns of the production stage. The maximum local velocity decreased from 0.009 Å/ps before retention to 0.008 Å/ps after retention. The velocity profile showed a parabolic-like distribution, with the largest values near the channel center and substantially smaller values near the boundaries. This spatial pattern arose because the Pt wall atoms imposed a no-slip-like condition at the interface, reducing the translational motion of nearby water molecules and the reduced construct. The overall reduction in maximum velocity after retention indicated the suppression of the reduced model's translational mobility upon contact with the rough Pt region. This dynamic response complemented the density increase and supported the interpretation of greater localization within the nanochannel.

**Fig. 5:**
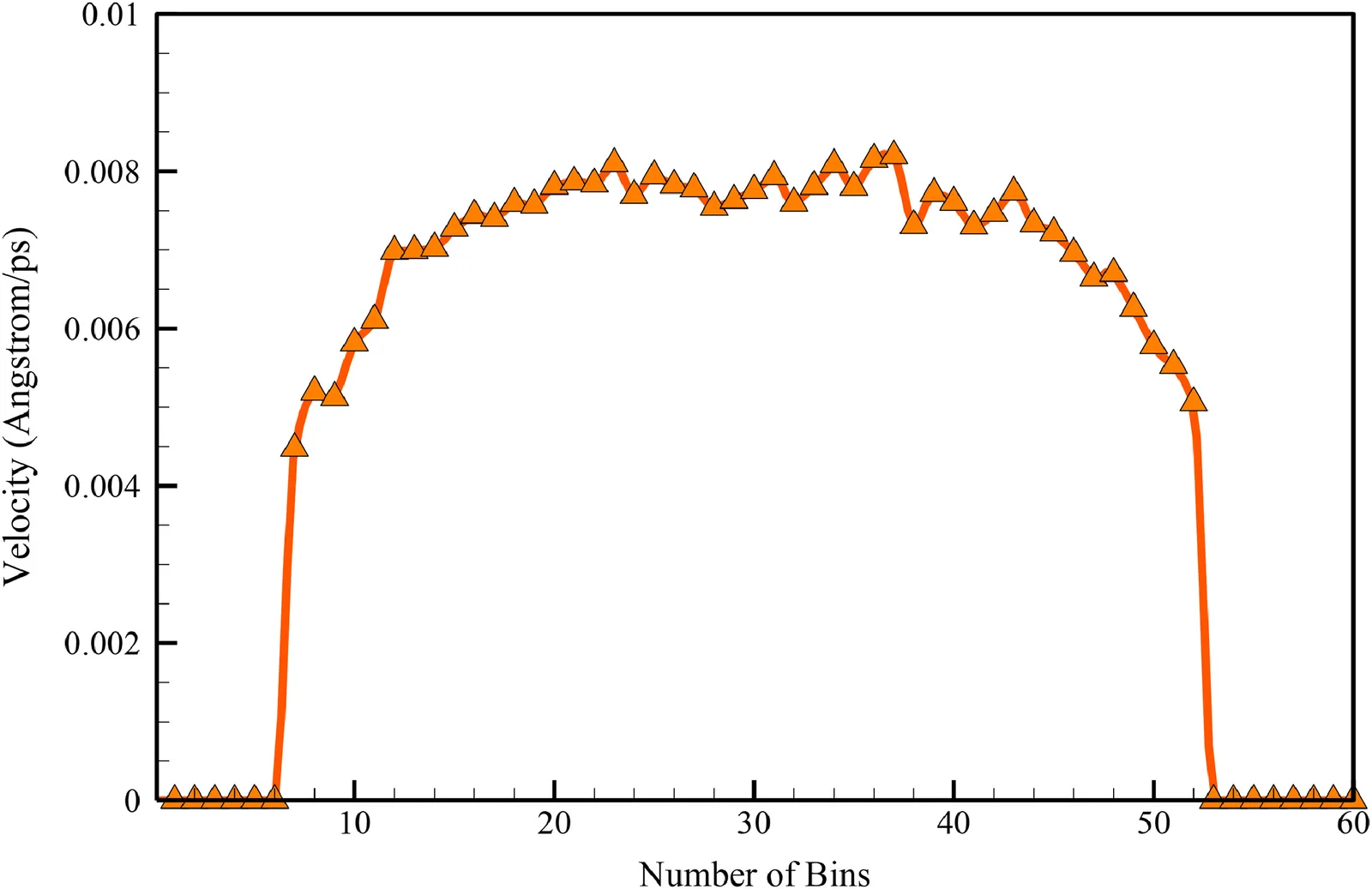
Velocity profile of the H₂O–reduced-model system after 20 ns of force-driven production.

Fig. 6 presents the local T profile during the force-driven production stage. For the 35 Å roughness case, the maximum local T decreased from 437.28 K before retention to 409.11 K after retention (Table 3). The elevated local Ts relative to the equilibration value of 300 K arise from the conversion of externally imposed mechanical work into kinetic energy of atoms during the nonequilibrium driving process. The subsequent decrease in maximum local T after retention shows dissipation of excess kinetic energy as the reduced construct's translational motion is restricted by the rough Pt geometry. The T reduction therefore accompanied reduced mobility and increased spatial localization of the reduced model.

**Fig. 6:**
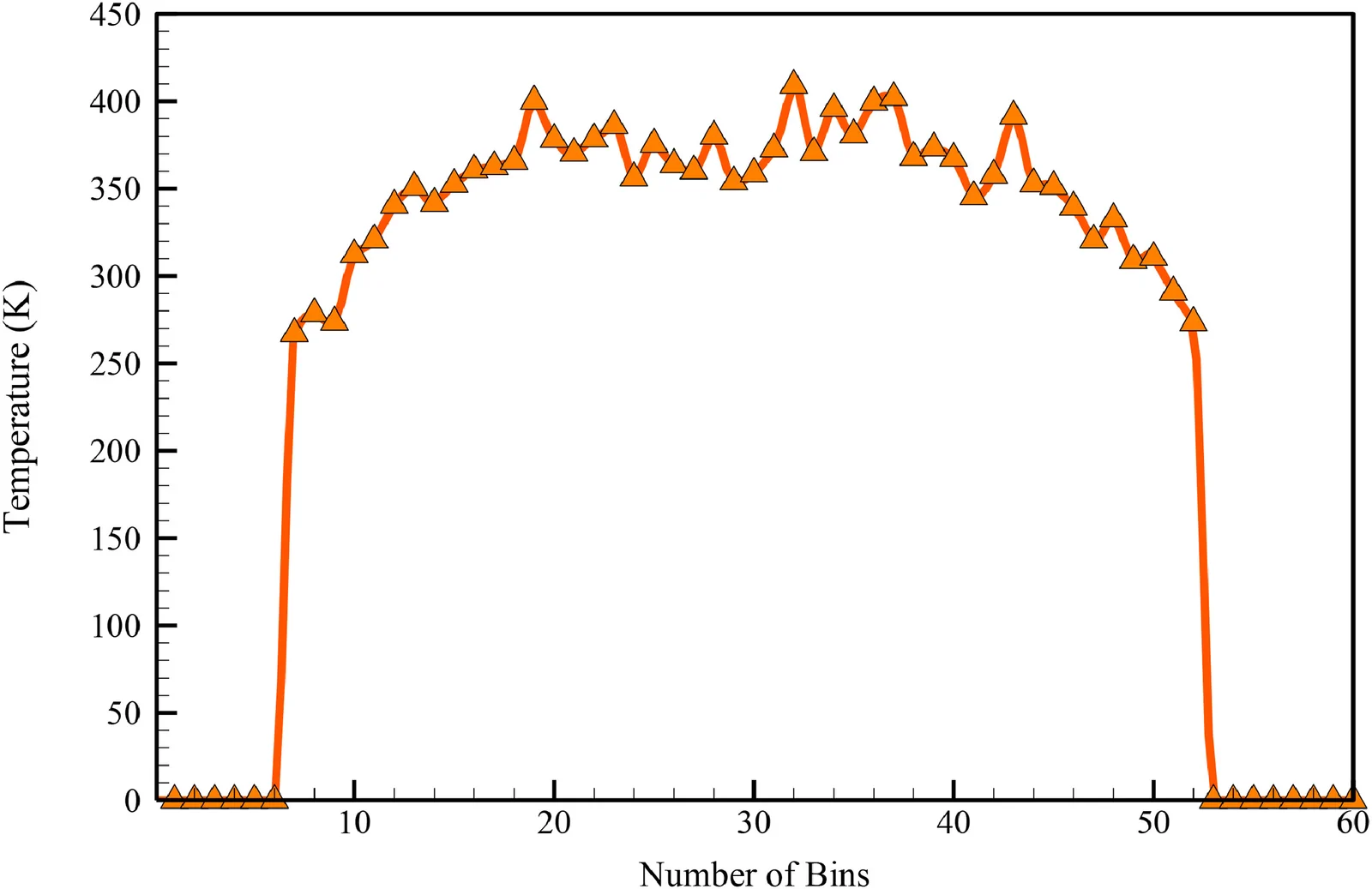
Local T profile of the H₂O–reduced-model system after 20 ns of force-driven production.

**Table 3:** Maximum density, velocity, and local T before and after retention of the reduced viral model under the 35 Å roughness condition.

System Type	Max D (atom/Å^3^)	Max V (Å/ps)	Max T (K)
**Before virus trapping**	0.174	0.009	437.28
**After virus trapping**	0.181	0.008	409.11

Table 3 summarizes the maximum density, velocity, and local T before and after retention of the reduced model under 35 Å roughness condition, providing a direct quantitative comparison of three kinetic descriptors. The coordinated increase in density, decrease in velocity, and decrease in T collectively characterized the force-driven retention of the reduced construct at this roughness size.

Collectively, the increase in maximum density (before and after virus trapping) from 0.174 to 0.181 atom/Å³ and the decrease in maximum velocity from 0.009 to 0.008 Å/ps revealed a coordinated response: the reduced construct became more spatially localized and kinetically restricted as it interacted with the rough Pt surface. The T decrease further supported this interpretation, as the kinetic energy dissipated through contact with the wall geometry contributes to the observed thermal relaxation. These three descriptors provided a consistent picture of the reduced model's force-driven retention behavior within the simulated nanochannel.

### 3.3 Effect of atomic roughness on confinement and retention of the reduced model

After establishing the retention behavior for a single roughness size, the investigation was extended to examine how varying the roughness geometry affected confinement of the reduced construct. Fig. 7 illustrates the initial and final configurations of the reduced 4-nm model during the force-driven production stage. The snapshots show the reduced construct displacing from its initial position toward the rough Pt region and then remaining localized within the nanochannel. We compared four roughness sizes (35, 37, 40, and 43 Å) while holding all other simulation parameters constant, isolating the effect of surface geometry on the reduced model's confinement.

**Fig. 7:**
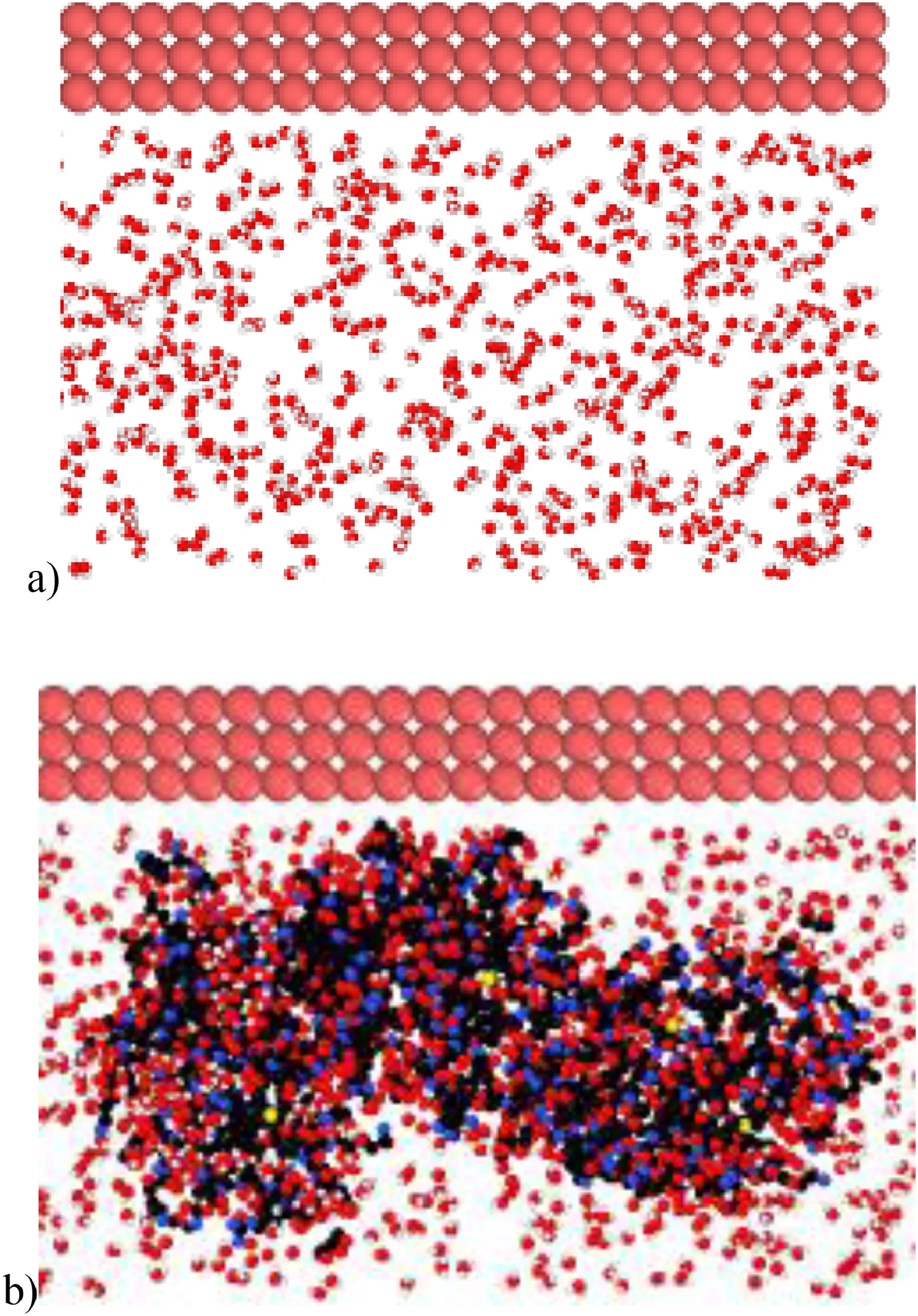
Representative initial and final configurations of the reduced 4-nm rabies-virus model during the force-driven retention process.

Fig. 8 and Table 4 summarize the maximum density, velocity, and local T of the reduced model as functions of atomic roughness size. As the roughness size increased from 35 to 40 Å, the maximum density increased from 0.181 to 0.187 atom/Å³, while the maximum velocity decreased from 0.008 to 0.006 Å/ps. The maximum local T also decreased from 409.11 to 399.17 K. These coordinated trends indicated that, within this range, larger roughness features enhanced the spatial localization of the reduced construct and more effectively suppressed its translational mobility. The physical origin of this behavior lies in the geometric match between the reduced model and the roughness features: as the roughness size increased toward 40 Å, the Pt irregularities created deeper, more accommodating cavities that increased the contact area with the reduced construct, thereby strengthening the confining effect.

**Fig. 8:**
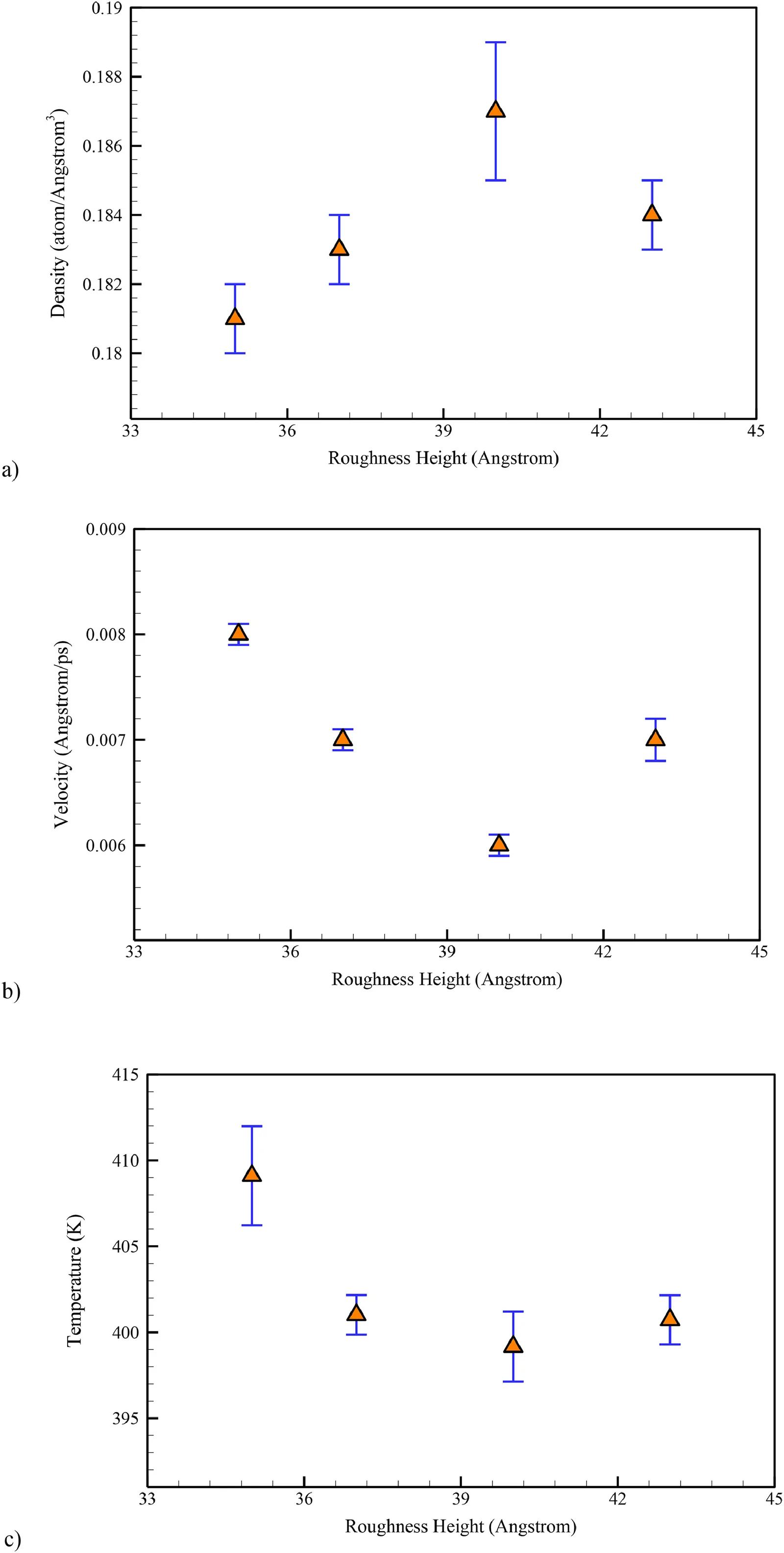
Maximum values of (a) density, (b) velocity, and (c) local T of the H₂O–reduced-model system as functions of atomic roughness size.

**Table 4:** Maximum density, velocity, and local T of the reduced model as functions of atomic roughness size.

Atomic roughness size(Å)	Max density (atom/Å^3^)	Max velocity (Å/ps)	Max T (K)
**35**	0.181±0.001	0.008±0.001	409.11±2.88
**37**	0.183±0.001	0.007±0.001	401.02±1.15
**40**	0.187±0.002	0.006±0.001	399.17±2.03
**43**	0.184±0.001	0.007±0.002	400.73±1.43

When the roughness size was further increased to 43 Å, the response reversed: the maximum density decreased to 0.184 atom/Å³, the maximum velocity increased to 0.007 Å/ps, and the maximum T increased slightly to 400.73 K. This reversal indicated that the roughness effect on the reduced construct was non-monotonic. Beyond 40 Å, the roughness features became too large relative to the reduced model's characteristic size, reducing the effective contact area and weakening geometric confinement. The reduced construct can no longer fully engage with the roughness cavities, leading to less efficient localization. The 40 Å configuration therefore produced the strongest confinement response of the reduced model among the four simulated cases, representing the roughness size at which the geometric compatibility between the Pt features and the reduced construct was maximized.

### 3.4 Retained volume and reduced-model–nanochannel interaction

To further quantify retention behavior, we calculated the surface-mesh volume associated with the retained reduced model using the Construct Surface Mesh module in OVITO (Stukowski, 2010). As shown in Fig. 9a, this volume increased from 24,691 Å³ at a roughness size of 35 Å to 31,118 Å³ at 40 Å. At 43 Å, the retained volume decreased to 24,987 Å³. The maximum retained volume at 40 Å showed the strongest spatial confinement of the reduced construct. This variation confirmed the non-monotonic response observed from the density and velocity descriptors and indicated that roughness size changes in the presence of different virus types directly affect adsorption of the target molecule (virus), as expected in actual applications.

**Fig. 9:**
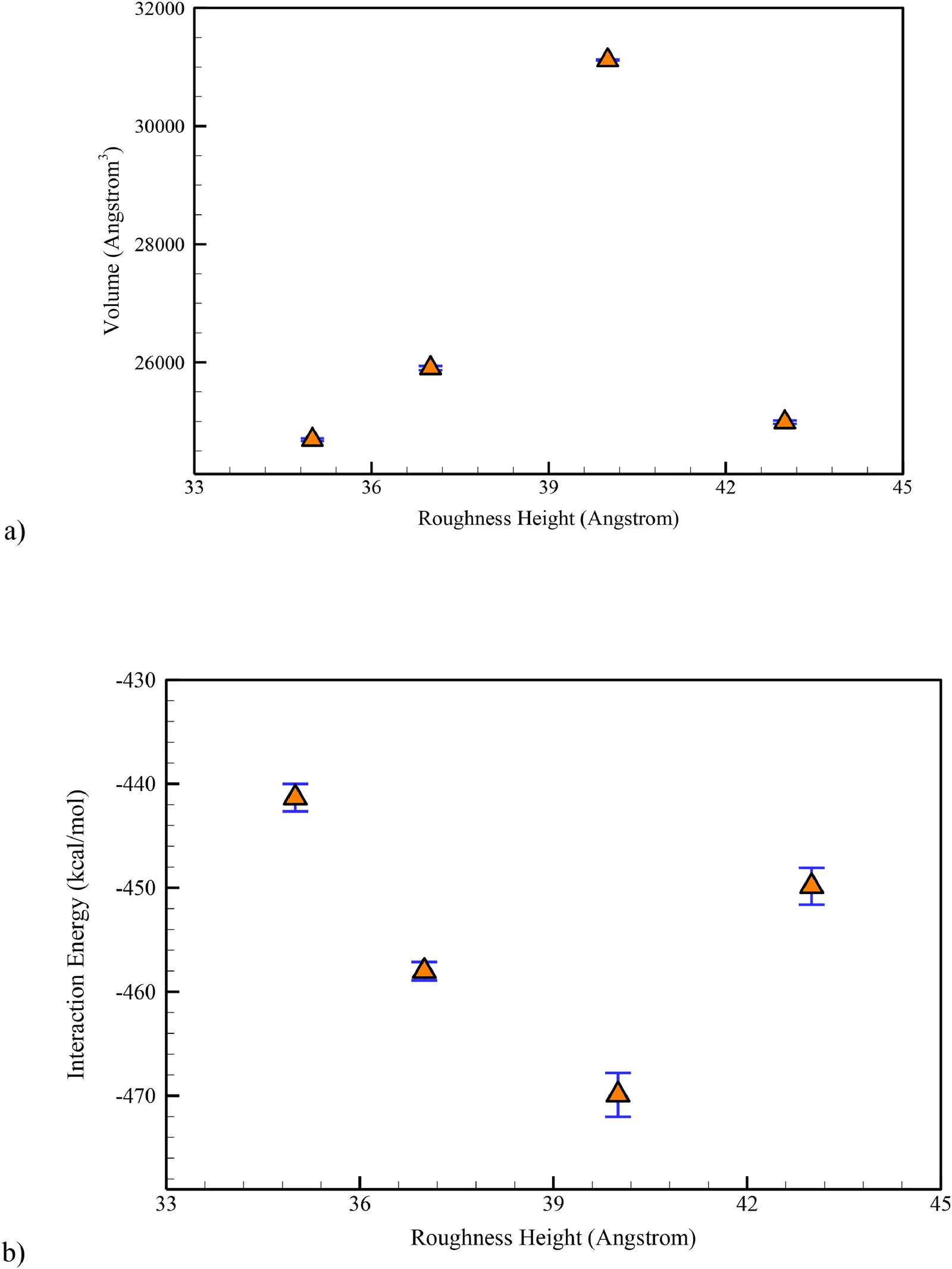
(a)Retained surface-mesh volume of the reduced viral model and (b) reduced-model–Pt interaction energy as functions of atomic roughness size.

We calculated the simulation-defined retained fraction by counting the reduced-model structures residing within the predefined roughness region at the end of the 20 ns production stage, relative to the total number of modeled structures. This fraction reached 95% for the 40 Å configuration, representing a simulation-specific retained fraction of the reduced 4-nm construct within the finite atomistic cell. The high retained fraction at 40 Å further corroborated the optimal geometric compatibility between the reduced construct and the roughness features at this size. We calculated the reduced-model–Pt interaction energy as the energy difference between the reduced-model–wall system after retention and the isolated arrangement, using the "compute group/group" command in LAMMPS (Simulator, Molecular Massively Parallel, 2003). This energy quantifies the strength of interfacial contact between the reduced construct and the Pt surface.

As shown in Fig. 9b, the interaction energy changed from −441.33 kcal/mol at 35 Å to −458.02 kcal/mol at 37 Å, reaching its most negative value of −469.91 kcal/mol at 40 Å. Within the adopted classical force-field description, a more negative interaction energy indicated a more energetically favorable contact between the reduced model and the Pt surface. The progressive strengthening of interaction from 35 to 40 Å reflects increasing contact area as the roughness features better matched the size of the reduced construct, allowing more Pt atoms to interact with the reduced model simultaneously. At 43 Å, the interaction energy became less negative (−449.85 kcal/mol), consistent with the reduced contact area when the roughness features exceeded the optimal geometric match. This trend paralleled the retained volume behavior and reinforced the interpretation that an appropriate geometrical relationship between the reduced construct and the roughness region was required to maximize contact while preserving access to the retention site. The nonequilibrium interaction energies served as comparative descriptors of the reduced model across the investigated roughness conditions.

To visualize the retention outcome, Fig. 10 presents a representative snapshot of the reduced 4-nm model after 20 ns of force-driven production. The reduced construct was localized within the rough Pt region, confirming that the wall's geometric features successfully confined the reduced model under the applied external force. This structural snapshot provided direct visual evidence that complemented the quantitative descriptors above: the retained volume, interaction energy, and 95% retained fraction all corresponded to the configuration shown in this figure, where the reduced model resides within the predefined roughness region of the nanochannel. The spatial correspondence between the reduced construct and Pt roughness features at 40 Å (the strongest confinement case) was particularly evident, as the reduced model appeared to conform to cavities created by surface irregularities, maximizing interfacial contact area.

**Fig. 10:**
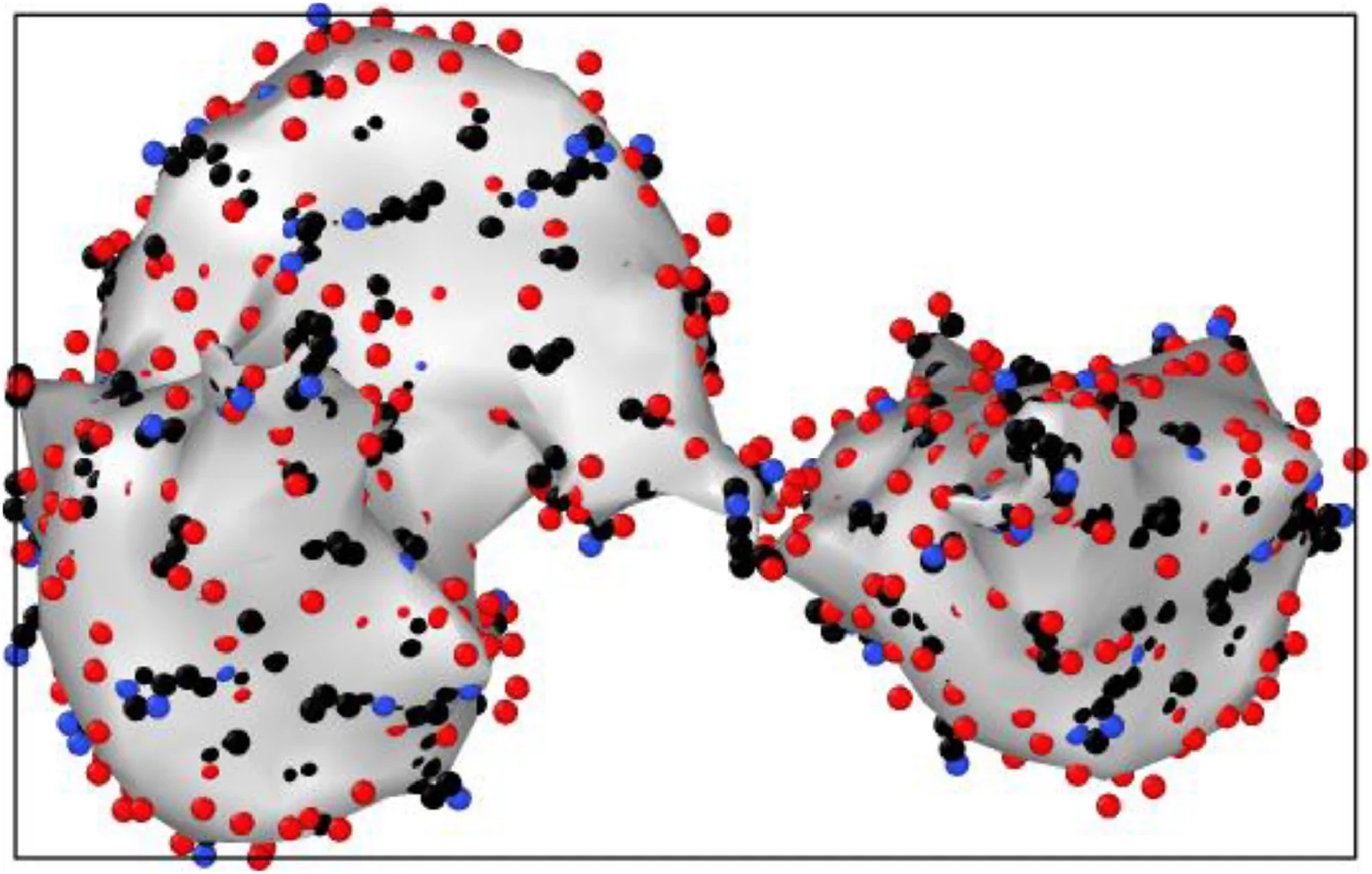
Representative reduced 4-nm model retained after 20 ns of force-driven production.

### 3.5 Energetic response of the reduced model

Fig. 11 and Table 5 present additional energetic descriptors calculated for the reduced model: a free-energy descriptor and the internal interaction energy. We computed the free-energy descriptor using the ΔH − TΔS relation, where H, T, and S represent the enthalpy, temperature, and entropy, respectively. The equilibrium phase of modeled samples served as the reference state (H₀ and S₀) in the free-energy formalism, while the final H, T, and S values were obtained after the retention process of the reduced model. As shown in Fig. 11a, the free-energy descriptor increased from 12.39 kcal/mol at 35 Å to 14.88 kcal/mol at 37 Å, reached 18.92 kcal/mol at 40 Å, and then decreased to 16.03 kcal/mol at 43 Å. Computationally, we calculated this energy type using the ΔH-TΔS relation, where H, T, and S represented the enthalpy, temperature, and entropy parameters, respectively. We used the equilibrium phase of the modeled samples as the reference state (H0 and S0) in the free-energy formalism calculation. Thus, the numerical values of the enthalpy and entropy parameters after 10 ns were taken as H0 and S0, respectively. We calculated the final H, T, and S values after the virus-trapping process in the final MD step under the NVE ensemble. Computationally, the system's enthalpy is defined as H=E+PV, where E is the sum of kinetic and potential energies, P is the instantaneous pressure (computed from the virial and kinetic contributions), and V is the simulation box volume; this was a global, extensive thermodynamic quantity updated each timestep from current atomic positions, velocities, and stresses.

**Fig. 11:**
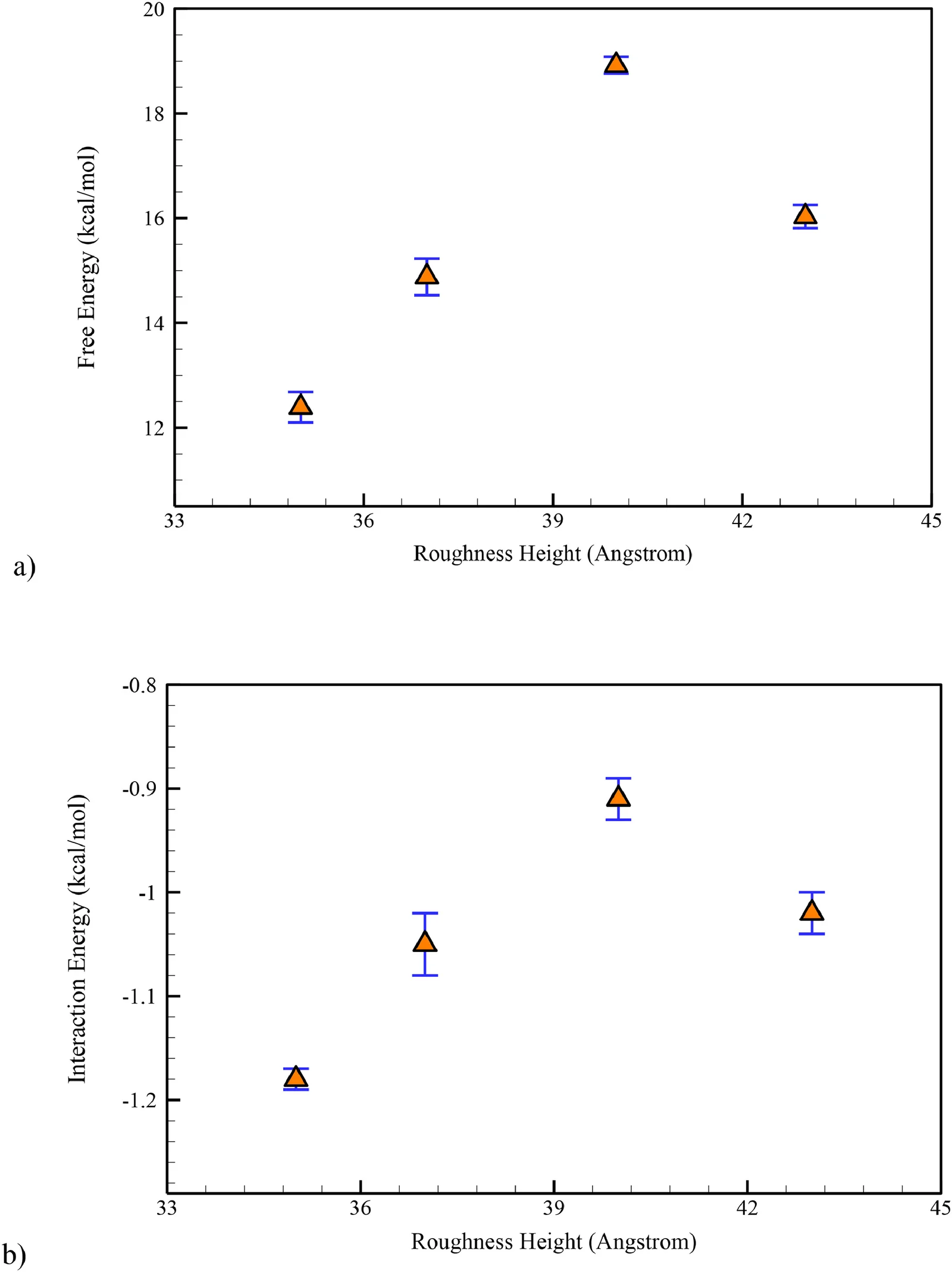
Variation in (a) the free-energy descriptor and (b) the internal interaction energy of the reduced viral model as functions of atomic roughness size.

**Table 5:** Retained volume, reduced-model–Pt interaction energy, free-energy descriptor, and internal interaction energy of the reduced viral model as functions of atomic roughness size.

Atomic roughness size(Å)	Volume (Å^3^)	Interaction Energy (kcal/mol)	Free Energy (kal/mol)	Interaction Energy (kcal/mol)
**35**	24691±23	-441.33±1.32	12.39±0.29	-1.18±0.01
**37**	25903±36	-458.02±0.89	14.88±0.35	-1.05±0.03
**40**	31118±11	-469.91±2.11	18.92±0.16	-0.91±0.02
**43**	24987±29	-449.85±1.77	16.03±0.22	-1.02±0.02

Furthermore, entropy was calculated using a per-atom pair-entropy fingerprint based on the local radial distribution function around each atom. The positive ΔG values reflected the combined contributions of the imposed external driving force, the confinement-induced structural rearrangement of the reduced construct, and the entropy variation associated with spatial localization. As the reduced model was driven into the rough Pt region, the external work performed on the system was converted into internal structural changes and entropic reduction due to the loss of translational freedom. The maximum free-energy descriptor at 40 Å corresponded to the roughness size at which the reduced construct underwent the greatest structural rearrangement, consistent with the strongest confinement observed in the density, velocity, and volume analyses. The decrease at 43 Å showed weaker confinement when the geometric match was lost.

The internal interaction energy of the reduced model, shown in Fig. 11b, changed from −1.18 kcal/mol at 35 Å to −1.05 kcal/mol at 37 Å and −0.91 kcal/mol at 40 Å, before increasing to −1.02 kcal/mol at 43 Å. The internal energy became less negative as the roughness size increased toward 40 Å, indicating that the internal structure of the reduced construct becomes less energetically favorable (a consequence of structural deformation and reorganization imposed by stronger confinement). At 40 Å, where the reduced model experienced the greatest spatial restriction, the internal interaction energy reached its least negative value (−0.91 kcal/mol), corresponding to the maximum structural perturbation. The slight recovery at 43 Å (−1.02 kcal/mol) showed reduced confinement when the roughness features exceeded the optimal size. These trends show that the internal energetic state of the reduced construct is sensitive to confinement geometry and varies non-monotonically with roughness size, consistent with all previous descriptors.

Taken together, five complementary descriptors (density, velocity, local T, retained volume, and interaction energy) converged on a consistent physical picture: retention of the reduced 4-nm model in the Pt nanochannel depended non-monotonically on atomic roughness size, with the strongest confinement at 40 Å. This behavior arose from geometric compatibility between the reduced construct and the roughness features, maximizing interfacial contact area and associated interaction energy at this size.

## 4 Conclusion

MD simulations were employed to investigate how rectangular atomic-scale roughness regulates the force-driven transport and retention of a reduced 4-nm model in a water-filled Pt nanochannel. The simulated construct, based on reported rabies-virus structural information, had a characteristic size of approximately 4 nm and reproduced the scale and atomic composition of a biological nanoparticle within a confined nanofluidic environment. The results therefore characterize comparative confinement mechanisms of the reduced model under the stated force-field and geometric conditions.

The initial 10 ns NVT equilibration produced a thermally relaxed configuration, as evidenced by stabilized potential energy and short-range ordering of the aqueous phase consistent with the TIP3P liquid structure. During the 20 ns production stage, the external driving force drove the reduced construct toward the rough Pt region, where the wall's geometric irregularities impeded further translational motion. Increasing the roughness size from 35 to 40 Å systematically enhanced the localization of the reduced model: the maximum density increased from 0.181 to 0.187 atom/Å³, the maximum velocity decreased from 0.008 to 0.006 Å/ps, and the retained surface-mesh volume increased from 24,691 to 31,118 Å³. In parallel, the reduced-model–Pt interaction energy became progressively more negative, changing from −441.33 to −469.91 kcal/mol, reflecting the increasing interfacial contact area as the roughness cavities became better matched to the size of the reduced construct. The simulation-defined retained fraction reached 95% at 20 ns for the 40 Å configuration, representing the strongest confinement of the reduced model among the four examined geometries.

Beyond 40 Å, the confinement response weakened. At 43 Å, the retained volume decreased to 24,987 Å³, the maximum velocity increased to 0.007 Å/ps, and the reduced-model–Pt interaction energy became less negative at −449.85 kcal/mol. The internal interaction energy of the reduced construct followed the same non-monotonic trend, reaching its least negative value at 40 Å (−0.91 kcal/mol) and recovering partially at 43 Å (−1.02 kcal/mol). These coordinated changes across five complementary descriptors (density, velocity, local T, retained volume, and interaction energy) show that the influence of roughness size on the reduced model is non-monotonic, with 40 Å representing the geometric condition that maximizes compatibility between the reduced construct and Pt surface features. The physical origin of this non-monotonic behavior lies in the geometric match between the reduced model and the roughness cavities: at 40 Å, the cavities accommodate the reduced construct effectively, maximizing contact and confinement; at larger roughness sizes, the cavities exceed the construct dimensions, reducing the effective contact area and weakening the retention.

Increasing the roughness size to 43 Å did not further improve retention. The retained volume decreased to 24,987 Å³, the maximum velocity increased to 0.007 Å/ps, and the reduced-model–Pt interaction energy became less negative at −449.85 kcal/mol. These coordinated changes demonstrated that the influence of roughness was non-monotonic and that 40 Å was the best-performing roughness size among four examined configurations. This result should not be interpreted as a universal optimum for full-size rabies viruses or practical nanochannels, and this structural parameter can vary across biological systems.

Overall, the simulations show that nanoscale surface morphology regulates the localization of reduced biological constructs through coupled geometrical confinement and interfacial interactions. The findings provided an atomistic-level understanding of how roughness size controlled the force-driven retention of a 4-nm construct in a Pt nanochannel, offering a computational framework for investigating roughness-dependent confinement in nanofluidic environments. The reported retained fraction, energetic values, and optimal geometry apply exclusively to the present 4-nm reduced model under the specific simulation conditions adopted. Extending these findings to quantitative prediction of natural rabies-virion capture will require full-scale or multiscale viral representations, longer sampling periods, deformable channel surfaces, independent simulations, and experimental validation.

## Declaration of AI use

During the preparation of this manuscript, the authors used ChatGPT (OpenAI) to improve the readability and language quality of the text. The authors reviewed and edited the generated content and take full responsibility for the accuracy and integrity of the final manuscript.

## CRediT authorship contribution statement

**Narinderjit Singh Sawaran Singh:** Formal analysis, Data curation, Conceptualization, Writing – original draft. **Murtaja Satea:** Formal analysis, Data curation, Conceptualization, Writing – original draft. **Ali Jawad Kadhum:** Methodology, Investigation, Funding acquisition, Formal analysis, Data curation. **Ibrahm Mahariq:** Methodology, Investigation, Funding acquisition, Formal analysis, Data curation. **Hemn A.H. Barzani:** Writing – original draft, Writing – review & editing. **Baydaa Abed Hussein:** Writing – original draft, Writing – review & editing. **Mina D. Jumaah:** Software, Validation. **Mahmut Taner:** Software, Validation. **Soheil Salahshour:** Project administration, Formal analysis, Writing – original draft, Writing – review & editing. **A. Rahimi:** Project administration, Formal analysis, Writing – original draft, Writing – review & editing.

## Declaration of competing interest

The authors declare that they have no known competing financial interests or personal relationships that could have appeared to influence the work reported in this paper.

## Data Availability

No data was used for the research described in the article.

## References

[bib0001] Albertini A.A.V., Schoehn G., Weissenhorn W., Ruigrok R.W.H. (2008). Structural aspects of rabies virus replication. Cell. Mol. Life Sci..

[bib0041] Alkhafaji A., Alaloosi W., Singh N.S.S., Barzani H.A.H., Taner M., Salahshour S., Sahramaneshi H. (2026). Effects of external electric field and heat flux on the stability of the SARS-CoV-2 main protease in the vicinity of water/silver nanofluid using molecular dynamics simulation. South African J. Chem. Eng..

[bib0043] Alkhafaji A., Zheoat A.M.A., Al-Dahhan M.R.H., Singh N.S.S., Haji B.S., Garalleh H.A.L., Taner M. (2026). Investigating the effect of variable electric-field amplitude and frequency on the interaction of water/silver nanofluid with the SARS-CoV-2 main protease: a molecular dynamics study. Res. Eng..

[bib0002] Bano I., Sajjad H., Shah A.M., Leghari A., Mirbahar K.H., Shams S., Soomro M. (2017). A review of rabies disease, its transmission and treatment. J. Anim. Health Prod..

[bib0003] Cai W., Sabetvand R., Abed A.M., Toghraie D., Hekmatifar M., Rahbari A., Yasin G., Abdulkadhim A.H., Smaisim G.F. (2022). Thermal analysis of hydration process in the vicinity of the copper matrix using molecular dynamics simulation for application in thermal engineering. Energy Rep..

[bib0004] Chen W., Zhang H., Wang J., Hu X. (2019). Flavonoid glycosides from the bulbs of Lilium speciosum var. gloriosoides and their potential antiviral activity against RSV. Chem. Nat. Compd..

[bib0005] Clark G.N.I., Cappa C.D., Smith J.D., Saykally R.J., Head-Gordon T. (2010). The structure of ambient water. Mol. Phys..

[bib0006] Daw M.S., Baskes M.I. (1984). Embedded-atom method: derivation and application to impurities, surfaces, and other defects in metals. Phys. Rev. B.

[bib0040] Gataa I.S., Hussein B.A., Sajadi S.M., Aljaafari H.A.S., Salahshour S., Baghaie S. (2025). The influence of initial temperature on the interactions of water/silver nanofluid with SARS virus using molecular dynamics simulation. Pow. Tech..

[bib0007] Guo Y., Song W., Dong Y., Wang X., Nie G., Li F. (2024). A poly aptamer encoded DNA nanocatcher informs efficient virus trapping. Nano Lett..

[bib0008] Hairer E., Lubich C., Wanner G. (2003). Geometric numerical integration illustrated by the störmer–verlet method. Acta Numer..

[bib0009] Hanwell M.D., Curtis D.E., Lonie D.C., Vandermeersch T., Zurek E., Hutchison G.R. (2012). Avogadro: an advanced semantic chemical editor, visualization, and analysis platform. J. Cheminformat..

[bib0039] Hashim H.O., Al-Shuhaib J.M.B., Mohammed M.K., Al-Shuhaib M.B.S. (2025). Targeting Monkeypox virus methyltransferase: Virtual screening of natural compounds from Middle-Eastern medicinal plants. Mol. Bio..

[bib0010] Hayek S.I. (2003). Mechanical vibration and damping. Digit. Encycl. Appl. Phys..

[bib0011] Hilbert S., Hänggi P., Dunkel J. (2014). Thermodynamic laws in isolated systems. Phys. Rev. E.

[bib0012] Hu W., Jiang D., Chen C., Zhou Y., Zhang S., Zhang N., Wang L. (2025). Advances in nanochannel electrochemical sensing technology for food safety detection. Trends Food Sci. Technol..

[bib0013] Jia Y., Fu B., Dong L., Zhao M. (2023). Sweet syndrome induced by SARS-CoV-2 vaccines: a systematic review of patient-report studies. Hum. Vaccines Immunother..

[bib0014] Jin J., Wu M., Ouyang A., Li K., Chen C. (2025). A novel dynamic hill cipher and its applications on medical IoT. IEEE Internet Thing. J..

[bib0015] Jones J.E. (1924). Proceedings of the Royal Society of London. Series A, Containing Papers of a Mathematical and Physical Character.

[bib0016] Jorgensen W.L., Chandrasekhar J., Madura J.D., Impey R.W., Klein M.L. (1983). Comparison of simple potential functions for simulating liquid water. J. Chem. Phys..

[bib0017] Klauda J.B., Wu X., Pastor R.W., Brooks B.R. (2007). Long-range lennard-Jones and electrostatic interactions in interfaces: application of the isotropic periodic sum method. J. Phys. Chem. B.

[bib0018] Lopez N.P., Martinez J.F.I., Vosburg C., Rajotte E.G., Rosa C., Terrones M. (2024). Effective plant virus enrichment using carbon nanotubes and microfluidics. J. Virol. Method..

[bib0019] Martínez L., Andrade R., Birgin E.G., Martínez J.M. (2009). PACKMOL: a package for building initial configurations for molecular dynamics simulations. J. Comput. Chem..

[bib0020] Mayo S.L., Olafson B.D., Goddard W.A. (1990). DREIDING: a generic force field for molecular simulations. J. Phys. Chem..

[bib0021] Plimpton S. (1995). Fast parallel algorithms for short-range molecular dynamics. J. Comput. Phys..

[bib0022] Plimpton S., Pollock R., Stevens M. (1997). Particle-mesh ewald and rRESPA for parallel molecular dynamics simulations. PP.

[bib0023] Plimpton S.J., Thompson A.P. (2012). Computational aspects of many-body potentials. MRS Bull..

[bib0024] Press W., Teukolsky S., Vetterling W., Flannery B. (2007). Numerical recipes: The art of scientific computing.

[bib0025] Rappé A.K., Casewit C.J., Colwell K.S., Goddard W.A., Mason Skiff W. (1992). UFF, a full periodic table force field for molecular mechanics and molecular dynamics simulations. J. Am. Chem. Soc..

[bib0026] Riedel C., Vasishtan D., Pražák V., Ghanem A., Conzelmann K.-K., Rümenapf T. (2019). Cryo-EM structure of the rabies virus ribonucleoprotein complex. Sci. Rep..

[bib0027] Saldaña F., Stollenwerk N., Bidaurrazaga Van Dierdonck J., Aguiar M. (2024). Modeling spillover dynamics: understanding emerging pathogens of public health concern. Sci. Rep..

[bib0028] Shiohara A., Wojnilowicz M., Lyu Q., Pei Y., Easton C.D., Chen Y., White J.F. (2023). SARS-CoV-2 virus detection via a polymeric nanochannel-based electrochemical biosensor. Small.

[bib0029] Simulator, Molecular Massively Parallel (2003). https://docs.lammps.org/pair_style.html.

[bib0030] Simulator, Molecular Massively Parallel (2003). https://docs.lammps.org/compute_group_group.html.

[bib0031] Stukowski A. (2010). Visualization and analysis of atomistic simulation data with OVITO–the open visualization tool. Model. Simul. Mater. Sci. Eng..

[bib0032] Tipler, P.A., and G. Mosca. "Physics for scientists and engineers, volume 1: mechanics, oscillations and waves." (1999): 470-471. ISBN: 9780321035714.

[bib0033] Verlet L. (1967). Computer" experiments on classical fluids. I. Thermodynamical properties of lennard-Jones molecules. Phys. Rev..

[bib0034] Wang W., Li B., Wu Y., Li M., Ma S., Yan D., Li D. (2024). Macrophage-derived biomimetic nanoparticles for light-driven theranostics toward Mpox. Matter.

[bib0042] Xiao L., Basem A., Zhang Y., Jasim D.J., Salahshour S., Li Z., Toghraie D. (2024). A numerical study of the effect of variable heat flux on the stability and thermal behavior of SARS-COV-2 structure: A molecular dynamics approach. Case Studies Therm. Eng..

[bib0035] Xu K., Gao B., Li J., Xiang Y., Cao L., Zhao M. (2023). Clinical features, diagnosis, and management of COVID-19 vaccine-associated Vogt-Koyanagi-Harada disease. Hum. Vaccin. Immunother..

[bib0036] Zeng H., Zhang Y., Zhang M., Liu H., Liu J., Zhu L., Wang J., Guo W. (2025). A signal-enhanced lateral flow immunoassay based on core–shell quantum dots labeled antibody and antigen for sensitive detection of CP4-EPSPS protein. Microchem. J..

[bib0037] Zhang H.-Q., Cao B.-Z., Cao Q.-T., Hun M., Cao L., Zhao M.-Y. (2023). An analysis of reported cases of hemophagocytic lymphohistiocytosis (HLH) after COVID-19 vaccination. Hum. Vaccine. Immunother..

[bib0038] Zhang Y.X., Alizadeh A., Abed A.M., Nasajpour-Esfahani N., Smaisim G.F., Hadrawi S.K., Zekri H., Baghaei S., Esmaeili S., Wang M.X. (2023). Investigating the effect of size and number of layers of iron nanochannel on the thermal behavior and phase change process of calcium chloride/sodium sulfate hexahydrate with molecular dynamics simulation. J. Energy Storage.

